# Copper-67 radioimmunotheranostics for simultaneous immunotherapy and immuno-SPECT

**DOI:** 10.1038/s41598-021-82812-1

**Published:** 2021-02-11

**Authors:** Guiyang Hao, Tara Mastren, William Silvers, Gedaa Hassan, Orhan K. Öz, Xiankai Sun

**Affiliations:** 1 Department of Radiology, University of Texas Southwestern Medical Center, Dallas, TX 75390 USA; 2 Advanced Imaging Research Center, University of Texas Southwestern Medical Center, Dallas, TX 75390 USA

**Keywords:** Biotechnology, Cancer, Biomarkers, Oncology, Chemistry

## Abstract

Copper-67 (t_1/2_ = 2.58 days) decays by β^−^ ($\text{E}_{{\upbeta^{-}\text{max}}}$: 562 keV) and γ-rays (93 keV and 185 keV) rendering it with potential for both radionuclide therapy and single-photon emission computed tomography (SPECT) imaging. Prompted by the recent breakthrough of ^67^Cu production with high specific activity, high radionuclidic purity, and sufficient quantities, the interest in the theranostic potential of ^67^Cu has been rekindled. This work addresses the practicability of developing ^67^Cu-labeled antibodies with substantially improved quality for cancer radioimmunotheranostics. Proof of concept is demonstrated with pertuzumab, a US-FDA-approved monoclonal antibody for combination therapies of HER2-positive breast cancer. With an average number of 1.9 chelators coupled to each antibody, we achieved a two-order of magnitude increase in radiolabeling efficiency compared to literature reports. In a preclinical therapeutic study, mice (n = 4–7/group) bearing HER2^+^ xenografts exhibited a ^67^Cu-dose dependent tumor-growth inhibition from ^67^Cu-labeled-Pertuzumab co-administered with trastuzumab. Furthermore, greater tumor size reduction was observed with ^67^Cu-labeled-pertuzumab formulations of higher specific activity. The potential of SPECT imaging with ^67^Cu radiopharmaceuticals was tested after ^67^Cu-labeled-Pertuzumab administration. Impressively, all tumors were clearly visualized by SPECT imaging with ^67^Cu-labeled-Pertuzumab even at day 5 post injection. This work demonstrates it is practical to use ^67^Cu radioimmunoconjugates for cancer radioimmunotheranostics.

## Introduction

Represented by [^177^Lu]Lu-DOTA-TATE therapy for midgut neuroendocrine tumors and [^177^Lu]Lu-PSMA-617 therapy for metastatic castration resistant prostate cancer, targeted radionuclide therapy has recently drawn considerable attention with paradigm shifting impacts on cancer patient care. If necessary, the radionuclide can be directly used or replaced with a surrogate for nuclear imaging to stratify patients for precision treatment and simultaneously monitor the radiopharmaceutical delivery^1–5^. While many factors contribute to the success of ^177^Lu-enabled radionuclide therapy, the cost-effective availability of high quality ^177^Lu at sufficient quantities has played an essential role^6^. For instance, a sufficient amount of ^177^Lu (~ 0.37 TBq) can be produced from one irradiation to treat 300–500 patients^7^. Currently, the specific activity of produced ^177^Lu has reached the range of 0.74 ‒ 2.96 TBq (20–80 Ci)/mg, which is approaching its theoretical specific activity of 4.10 TBq (110.9 Ci)/mg^6^. In addition to its β^−^ emissions [$\text{E}_{{\upbeta^{-}\text{max}}}$: 497 keV (78.6%), 384 keV (9.1%), and 176 keV (12.2%)] for radiotherapy, ^177^Lu decays by γ-rays [E_γ_ = 113 keV (6.6%), 208 keV (11%)]^8^, which enables SPECT imaging and thus theranostic use of ^177^Lu radiopharmaceuticals. However, the branching ratios of ^177^Lu’s γ photons are suboptimal for SPECT imaging sensitivity. As such, replacing ^177^Lu with a positron emitter, such as ^68^Ga (t_1/2_ = 68 min), for positron emission tomography (PET) imaging prior to radiotherapy with ^177^Lu has become a common practice in nuclear medicine^9,10^. Although both are trivalent metal ions, ^68^Ga differs from ^177^Lu in their coordination chemistry. For the design of an ideal theranostic pair, an element with both therapeutic and diagnostic radioisotopes is preferred, such as yttrium (^86^Y/^90^Y), iodine (^123/124^I/^131^I), and copper (^64^Cu/^67^Cu).

With the mean β^−^-emission energy of 141 keV [$\text{E}_{{\upbeta^{-}\text{max}}}$: 562 keV], which is slightly higher than that of ^177^Lu, ^67^Cu has long been suggested for radiotherapy since 1980s^11,12^. The β^−^ emissions from ^67^Cu permit the irradiation of several layers of tumor cells and its shorter half-life (t_1/2_ = 2.58 d) is more optimal for patient care. In addition to ^67^Cu, copper has several other radioisotopes (^60^Cu, ^61^Cu, ^62^Cu, and ^64^Cu) that have been reported for the development of PET radiopharmaceuticals. Of them, ^64^Cu has been widely used for preclinical and clinical PET studies due to its moderate half-life (t_1/2_ = 12.7 h), low positron energy, and availability^13^. Given that copper radioisotopes are chemically identical, the same bifunctional chelators that have been developed for ^64^Cu radiopharmaceuticals can be used directly for ^67^Cu radiopharmaceuticals^13^. More importantly, PET imaging can be made readily available by simply swapping ^67^Cu with ^64^Cu in the same radiopharmaceutical when desired for pre-screen of patients for targeted ^67^Cu-radionulide therapy or dosimetry estimation. Surprisingly to some extent, in the reported nuclear medicine applications of ^67^Cu, its γ-emissions at 93 keV (16%) and 185 keV (49%)^14^ are largely neglected for SPECT imaging. Of note, the branching ratios of ^67^Cu’s γ-emissions are much higher than those of ^177^Lu’s, which would provide the much-needed imaging sensitivity.

Despite the potential for both imaging and therapy, the use of ^67^Cu for targeted radionuclide therapy has been hampered for decades by its limited supply and low specific activity. The production of ^67^Cu has been tested on a variety of nuclear reactions, such as ^68^Zn(p, 2p)^67^Cu, ^70^Zn(p, α)^67^Cu, ^68^Zn(γ, p)^67^Cu, and ^67^Zn(n,p)^67^Cu, in addition to heavy-ion fragmentation^15–25^. However, therapeutic quantities of ^67^Cu at high specific activity were not achieved until recently^21^. A major breakthrough was reported in the ^68^Zn(γ,p)^67^Cu reaction by irradiating a highly enriched ^68^Zn (98.97%) target with high energy γ rays produced by bremsstrahlung conversion of electrons from electron accelerator^26,27^. The specific activity of ^67^Cu so produced has reached over 5.55 GBq/μg (150 mCi/μg)^26^, which is a big leap as compared to that (0.69 GBq/μg or 18.6 mCi/μg) obtainable by conventional ^68^Zn(p, 2p)^67^Cu reaction^28^. Due to this breakthrough in ^67^Cu production, no-carrier-added ^67^Cu has been made available in large scales at the National Isotope Development Center^29^. Indeed, a clinical trial with ^67^Cu-Octreotate was reported in 2019^30^, and a few preclinical theranostic studies with ^67^Cu have been seen recently^31–33^. In this work, we evaluated the use of ^67^Cu produced by the electron accelerator method with the aim to reassess the role of ^67^Cu in radioimmunotherapy coupled with the up-to-date SPECT technology. A HER2 targeting antibody, pertuzumab, was used as a model antibody to construct the desired radioimmunotheranostic conjugate.

## Results

### Pertuzumab conjugation, ^67^Cu radiolabeling, and in vitro stability

**Figure 1:**
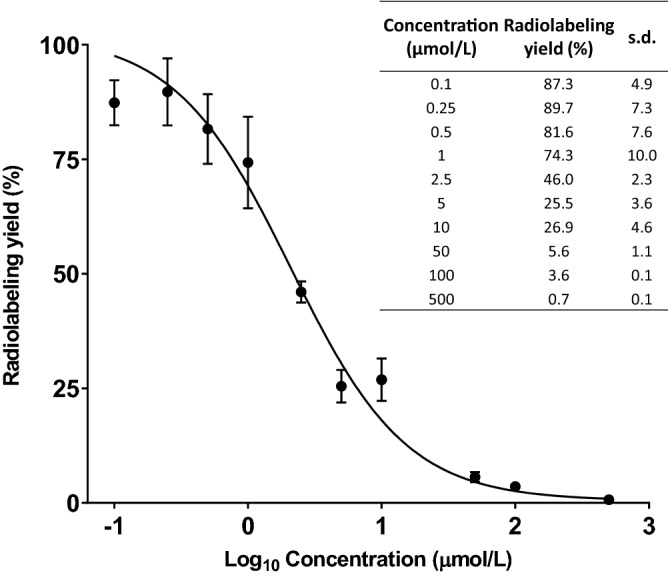
Determination of the average number of NOTA conjugated to each pertuzumab molecule with an isotopic dilution method. r^2^ = 0.9715; n = 3; s.d.: standard deviation. The figure was graphed with GraphPad Prism 7.0 (https://www.graphpad.com/scientific-software/prism/).

The conjugation of p-SCN-Bn-NOTA (2-S-(4-Isothiocyanatobenzyl)-1,4,7-triazacyclononane-1,4,7-triacetic acid) to pertuzumab (20:1 molar ratio) was performed at pH 8.5 for 3 h at room temperature, followed by purification by fast protein liquid chromatography (FPLC). Under the condition, the average number of NOTA chelators per pertuzumab molecule was determined to be 1.9 by the isotope dilution method (Fig. 1). We conducted a series of radiolabeling tests varying the amount of the antibody conjugate for labeling with a fixed amount (27 MBq) of ^67^Cu (Table 1) in order to obtain the highest achievable radiolabeling efficiency and specific activity of [^67^Cu]Cu-NOTA-Pertuzumab. By maintaining the amount of NOTA-Pertuzumab at 5 µg and above, the radiolabeling yield was virtually quantitative. When the conjugate amount dropped to 2 µg, 1 µg, and 0.5 µg, the radiochemical yield decreased to 54%, 32%, and 13%, respectively. Accordingly, the highest achievable specific activity was attained at 8.6 GBq/mg (or 230 mCi/mg) at the end of synthesis when the amount of conjugate was 1 µg. The radiopharmaceutical purity was over 99% after purification as determined by both radio-TLC and radio-FPLC. The in vitro stability of [^67^Cu]Cu-NOTA-Pertuzumab was found stable (> 97% intact) out to 7 days in rat serum, indicating no appreciable dissociation of ^67^Cu from the conjugate in the presence of serum proteins.

**Table 1:** Radiolabeling tests to obtain the highest achievable radiochemical yield and specific activity at the end of synthesis.

Reaction no	NOTA-Pertuzumab (µg)	^67^Cu (MBq)	Labeling yields (%)	Specific activity (GBq/mg)
1	125	27	> 99	0.2
2	25	27	> 99	1.1
3	5	27	> 99	5.4
4	2	27	54	7.3
5	1	27	32	8.6
6	0.5	27	13	7.0

### Immunoreactivity assay of [^67^Cu]Cu-NOTA-Pertuzumab and its specific binding to HER2

**Figure 2:**
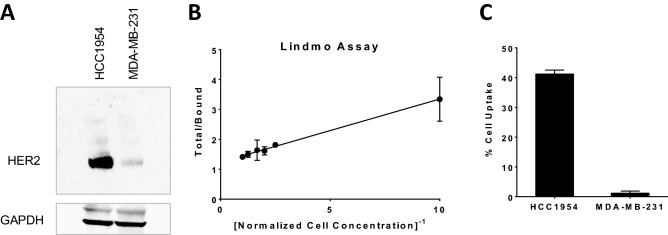
The immunoreactivity of pertuzumab was not compromised after NOTA conjugation and ^67^Cu-labeling. (**A**) Western blot analysis of HER2 positive HCC1954 and HER2 negative MDA-MB-231 cells (the blots are cropped for clarity. The full length blots at different exposure levels are presented in Supplementary Figure S1); (**B**) Lindmo assay of [^67^Cu]Cu-NOTA-Pertuzumab using HCC1954 cells; (**C**) Cell uptake assay of [^67^Cu]Cu-NOTA-Pertuzumab in HCC1954 and MDA-MB-231 cells. (**B**) was graphed with GraphPad Prism 7.0 (https://www.graphpad.com/scientific-software/prism/).

The immunoreactivity of [^67^Cu]Cu-NOTA-Pertuzumab was measured by the Lindmo assay using HER2 positive HCC1954 cells (Fig. 2A and Figure S1). The immunoreactive fraction of [^67^Cu]Cu-NOTA-Pertuzumab was determined to be 80.6%, which is comparable to previously reported ^67^Cu-labeled immunoconjugates^12,34^ or pertuzumab conjugates^35^. It indicates that the immunoreactivity of pertuzumab with HER2 was maintained when conjugated with NOTA at the molar ratio of 1:1.9 (Pertuzumab:NOTA) (Fig. 2B). The binding specificity of [^67^Cu]Cu-NOTA-Pertuzumab to HER2 receptors was examined by a comparative assay using HER2 positive HCC1954 and HER2 negative MDA-MB-231 cells. The % uptake of [^67^Cu]Cu-NOTA-Pertuzumab in the HCC1954 cells was found 33 times higher than that in MDA-MB-231 cells (*p* < 0.001) (Fig. 2C), further confirming that the immunoreactivity of [^67^Cu]Cu-NOTA-Pertuzumab was not compromised after NOTA conjugation and ^67^Cu labeling.

### Radioimmunotherapy with [^67^Cu]Cu-NOTA-Pertuzumab

**Figure 3:**
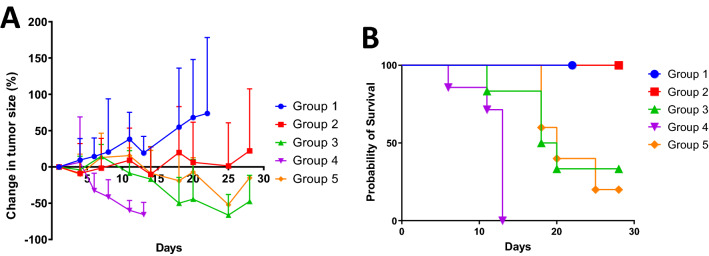
Results of radioimmunotherapy studies with [^67^Cu]Cu-NOTA-Pertuzumab in 5 groups of mice bearing HER2+ HCC1954 tumors. (**A**) Tumor size changes in percentage with respect to the size prior to treatment (n = 4–7); (**B**) Kaplan–Meier survival curves. Both figures were graphed with GraphPad Prism 7.0 (https://www.graphpad.com/scientific-software/prism/).

Recently, the co-presence of trastuzumab, another US-FDA approved therapeutic HER2 monoclonal antibody, was reported to enhance the accumulation of ^89^Zr-labeled Pertuzumab in HER2 positive tumors^35^, because a conformational change in HER2 that occurs upon trastuzumab binding might result in a greater exposure of the binding site to pertuzumab^36^. Therefore, we administered the same amount of trastuzumab to all the mice bearing HER2 positive HCC1954 xenografts for radioimmunotherapy trial. Mice were randomized into 5 treatment groups with [^67^Cu]Cu-NOTA-Pertuzumab: Group ***1*** as controls injected with 13.3 µg of pertuzumab to access the treatment effect directly from the antibody itself; Groups ***2***–***4*** as treatment groups injected with 3.7 MBq/3.3 µg (low dose), 7.4 MBq/6.7 µg (medium dose), and 14.8 MBq/13.3 µg (high dose) of [^67^Cu]Cu-NOTA-Pertuzumab, respectively, to evaluate the response to radiation dosing escalation; and Group ***5*** at the 7.4 MBq medium dose but co-injected with 133.3 µg of pertuzumab to evaluate the role of the specific activity of [^67^Cu]Cu-NOTA-Pertuzumab in the treatment response using a previously reported condition^34,37^. Shown in Fig. 3A and Figure S2, the tumor growth was halted by [^67^Cu]Cu-NOTA-Pertuzumab during the entire period of study as compared to the controls in all treatment groups including Group ***5***. Of note, a clear trend of tumor growth inhibition was observed even with the low dose of [^67^Cu]Cu-NOTA-Pertuzumab in Group ***2***, in which the tumor size remained almost the same out to 25 days. As the radiation dose escalated to the medium and high doses, which correspond to 2 times and 3 times of the low dose, respectively, tumor size reduction was observed almost immediately after the dose administration. Interestingly, we observed a better treatment outcome in Group ***3*** than in Group ***5***, both of which received the same amount of ^67^Cu-activity but the latter with 20 times lower specific activity.

### Radiation toxicity resulted from treatment with [^67^Cu]Cu-NOTA-Pertuzumab

All the mice in the control group (Group ***1***) survived to day 22 after the start of treatment but they were sacrificed due to the drastically increased tumor burden (Fig. 3B). Impressively, the mice in the low dose group (Group ***2***) experienced a weight loss (Figure S3) in the beginning but regained the weight back to normal as the control group within week 3, indicating that the single-dose injection of 3.7 MBq was tolerable. For the groups treated with escalated doses of 7.4 and 14.8 MBq, mice started to lose weight from day 4 post treatment in tandem with observed tumor burden reduction. The fact that the mice did not regain their weight back to normal indicates the unwanted systemic toxicity resulted from [^67^Cu]Cu-NOTA-Pertuzumab administered at the medium and high doses. Consequently, the mice in Group ***3*** (medium dose) showed 50% fatality rate on day 18 and an average survival of 20.5 days during the period of study, while those in Group ***4*** (high dose) only survived 11.7 days on average. Not surprisingly, Group ***5*** (medium dose but with low specific activity) exhibited a similar toxicity or survival trend to Group 3, with 50% fatality rate on day 20 and an average survival of 21.8 days.

### Immuno-SPECT/CT imaging with [^67^Cu]Cu-NOTA-Pertuzumab in HER2 positive xenografts

With its γ-emissions at 93 keV (16%) and 185 keV (49%), ^67^Cu allows immuno-SPECT imaging simultaneously with radioimmunotherapy with [^67^Cu]Cu-NOTA-Pertuzumab. In order to evaluate the theranostic potential of ^67^Cu, we performed SPECT/CT imaging in the mice (Groups ***2***–***5***) injected with [^67^Cu]Cu-NOTA-Pertuzumab for radioimmunotherapy. Recent technological advances have made quantitative SPECT imaging acquisition and image analysis possible with necessary corrections including but not limited to photon attenuation and scattering^38^. After development of an appropriate calibration file, we acquired quantitative SPECT imaging data of ^67^Cu in the unit of MBq/mL on a NanoSPECT/CT Plus scanner.

**Figure 4:**
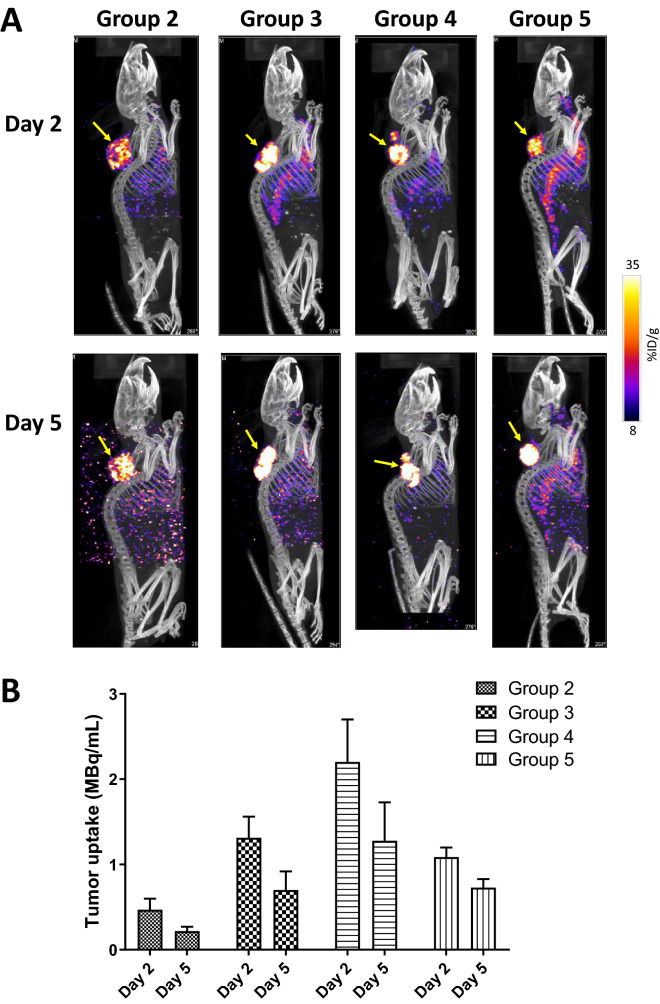
SPECT/CT imaging results. (**A**) Representative maximum intensity projection (MIP) SPECT/CT images of HCC1954 HER2+ tumor-bearing mice injected with [^67^Cu]Cu-NOTA-Pertuzumab (Group ***2***, ***3***, ***4***, and ***5***) at day 2 and 5 post the start of treatment (yellow arrows indicate the tumors); (**B**) Actual radioactivity concentration in tumors (MBq/mL) on Day 2 and 5 (without decay correction).

Shown in Fig. 4A, all the tumors were clearly visualized with [^67^Cu]Cu-NOTA-Pertuzumab by SPECT on both day 2 and day 5 post injection, even at the lowest injected dose (3.7 MBq). As the dose escalated from 3.7 MBq to 7.4 MBq (Group ***2*** vs. Group ***3***), the SPECT signal in the tumor was noticeably enhanced. In contrast, the signal intensity in the background tissues (e.g., liver) were much less than that in the tumor and decreased over time. Consequently, the tumor-to-background contrast became further enhanced over the time course, a highly desirable feature for clinical imaging data interpretation.

Quantitative tumor uptake from the SPECT imaging data are presented as absolute radioactivity concentration (µCi/mL) (Fig. 4B). Clearly, the dose escalation from Group 2 to Group 4 resulted in significantly increased radioactivity concentration in tumors (Group ***2***: 0.47 ± 0.13 MBq/mL at day 2 and 0.22 ± 0.05 MBq/mL at day 5; Group ***3***: 1.31 ± 0.25 MBq/mL at day 2 and 0.70 ± 0.22 MBq/mL at day 5; and Group ***4***: 2.20 ± 0.50 MBq/mL at day 2 and 1.28 ± 0.45 MBq/mL at day 5) (p < 0.05 between each data set at day 2 or day 5). A comparative quantitative analysis between Group ***3 ***and Group ***5*** showed that higher specific activity of [^67^Cu]Cu-NOTA-Pertuzumab led to noticeably increased radioactivity concentration in tumors on day 2 (Group ***3***: 1.31 ± 0.25 MBq/mL vs. Group ***5***: 1.09 ± 0.11 MBq/mL). Interestingly, the tumor radioactivity concentration difference between Group ***3 ***and Group ***5*** diminished at day 5 (Group ***3***: 0.70 ± 0.22 MBq/mL *vs.* Group ***5***: 0.73 ± 0.10 MBq/mL; *p* > 0.05). Reflected in the SPECT images, the higher specific activity Group ***3*** showed higher visual tumor uptake intensity than the lower specific activity Group ***5*** on day 2, but the difference disappeared on day 5.

## Discussion

Copper-67 has been long considered as an “ideal” radionuclide for radioimmunotherapy due to its highly desirable decay features^12,39^. However, a rather limited number of studies were reported in either preclinical investigation or clinical trial settings in radioimmunotherapy^40^, because of the low production yield, high cost, and limited availability of ^67^Cu^21^. The recent advancement in ^67^Cu production, represented by the ^68^Zn(γ,p)^67^Cu reaction, has elicited rapid developments of ^67^Cu radiopharmaceuticals for cancer theranostics with focuses on ^67^Cu labeled small molecules and peptides for radionuclide therapy^30–33^. Particularly important to antibody-based radiopharmaceuticals, ^67^Cu with high specific activity would revitalize the conventional radioimmunotherapy hampered by the low specific activity of ^67^Cu. Coupled with its SPECT imaging capability, ^67^Cu would allow the development of radioimmunotheranostics featuring both clinical potentials of radioimmunotherapy and immuno-SPECT.

Prior to the current source of ^67^Cu from the nuclear reaction of ^68^Zn(γ,p)^67^Cu, the obtainable specific activity of ^67^Cu from conventional production methods was in the range of 2–10 Ci/mg or 74–370 GBq/mg^37^. Accordingly, the specific activities of most ^67^Cu-labeled antibodies previously reported were in the range of 37–56 MBq (1–1.5 mCi)/mg of antibody^34,37^, which were too low to have practical applications in radioimmunotherapy, in particular when the antigen expression levels are low and thus can be readily saturated. Reported with the specific activity up to 150 Ci/mg or 5,550 GBq/mg^26^, the currently available source of ^67^Cu should be able to enhance the practicality of using ^67^Cu for radioimmunotherapy.

We performed the γ-spectroscopic analysis of the high specific activity ^67^Cu on a high purity germanium (HPGe) system. It confirmed that the ^67^Cu solution as-received was pure without any measurable radionuclidic contaminants two days after production (Figure S4). Because of the high quality of ^67^Cu in terms of high specific activity and high radionuclidic purity, we observed a remarkably high radiolabeling efficiency for radiolabeling of NOTA-Pertuzumab with the ^67^Cu, which is at least two order of magnitude greater than that reported in the literature with conventional ^67^Cu. Consequently, we were able to attain the specific activity of [^67^Cu]Cu-NOTA-Pertuzumab up to 8.6 GBq/mg (230 mCi/mg), two orders of magnitude higher than what reported for conventional ^67^Cu-labeled antibodies. Of note, with the molar ratio of NOTA-to-Pertuzumab at 1.9:1, the immunoconjugate of NOTA-Pertuzumab maintained over 80% immunoreactivity of its parent antibody, pertuzumab.

Given the fact that only a very limited number of radioimmunotherapy studies were previously reported with ^67^Cu^34,37^, there is a lack of references to the proper radiotherapeutic dose range of ^67^Cu radiopharmaceuticals. To provide a proof-of-concept, we set out to start with 3.7 MBq per mouse for a single intravenous administration, followed by dose escalation to 7.4 and 14.8 MBq. Indeed, the 3.7 MBq low dose treatment group showed a marked therapeutic effect measured by tumor-growth inhibition while all mice survived the treatment. The dose escalation to 7.4 and 14.8 MBq resulted in greater tumor burden reduction but a considerable toxicity emerged in the medium and high dose groups. As such, to overcome this toxicity issue, a dose fractionation strategy needs to be considered for future studies. To evaluate the added-value of the high specific activity of ^67^Cu, we conducted a comparative treatment study, in which Group ***3*** (1.10 GBq/mg) and Group ***5*** (53 MBq/mg) represent the currently achievable specific activity of ^67^Cu-labeled immunoconjugates and the conventional specific activity range (37–56 MBq/mg), respectively. We found that mice in Group ***3*** showed higher tumor accumulation at day 2, indicating high specific activity is beneficial to targeted delivery of radiation dose. This observation can be attributed to the fact that the antigen’s binding sites for [^67^Cu]Cu-NOTA-Pertuzumab are limited. Consequently, low specific activity [^67^Cu]Cu-NOTA-Pertuzumab might lead to low targeted delivery of ^67^Cu thus eliciting unwanted systemic toxicity because of the binding site saturation^41^. Indeed, we observed more effective tumor-growth inhibition for the high specific activity group (Group ***3***) than for its low specific activity counterpart. Of note, the specific activity of [^67^Cu]Cu-NOTA-Pertuzumab in Group ***3*** in this work was set conservatively at 1.10 GBq/mg, which was only about one seventh of the maximum achievable, in order to evaluate the practicality of radioimmunotherapy with [^67^Cu]Cu-NOTA-Pertuzumab. This is because a too high specific activity is not always desirable due to the fact that low expression of the targeted antigen may exist in non-target organs and passive non-specific binding may occur as well^42^.

Another major goal of this work was to impart the diagnostic value of ^67^Cu to radioimmunotherapy. Traditionally, the quantification capability of SPECT is limited as compared to PET largely due to the inherent physical detection sensitivity of γ-rays^43^. To date, the advancement of technology has rendered SPECT with the capability of absolute quantification of signals in regions of interest in terms of activity concentration (e.g., kBq/cc or equally μCi/mL) and normalized uptake, such as the standardized uptake value (SUV)^43–45^. Indeed, quantitative SPECT has been reported for disease diagnosis, patient stratification, radionuclide therapy dosimetry, and longitudinal follow-up imaging^43,46,47^. These advancements would potentially propel ^67^Cu into clinical applications, such as early diagnosis, dosimetry, prognosis, and dosage planning. To make it a reality, we managed to calibrate our small animal SPECT/CT scanner with ^67^Cu and generate the corresponding acquisition and analysis protocols so as to acquire and quantify ^67^Cu-SPECT images while performing the radioimmunotherapy studies. Shown in Fig. 4, we were able to perform immuno-SPECT imaging with [^67^Cu]Cu-NOTA-Pertuzumab along with quantitative data analysis, unequivocally indicating the feasibility of incorporating the diagnostic value of ^67^Cu into radiotherapies with ^67^Cu radiopharmaceuticals.

## Conclusion

The recent breakthrough of ^67^Cu production offers great opportunities to revitalize ^67^Cu radiopharmaceuticals by making available a reliable source of ^67^Cu with high specific activity, high radionuclidic purity, and sufficient quantity. Our work presented herein with [^67^Cu]Cu-NOTA-Pertuzumab demonstrates the potential as well as the practicality of using ^67^Cu for the design and development of cancer radioimmunotheranostics.

## Methods

### Chemicals and reagents

All chemicals, solvents, and reagents were purchased from Sigma-Aldrich and Fisher Chemical unless otherwise noted. Pertuzumab (Perjeta) and trastuzumab (Herceptin) were obtained from the pharmacy at University of Texas Southwestern Medical Center (UT Southwestern). p-SCN-Bn-NOTA was purchased from Macrocyclics Inc (Dallas TX). All aqueous solutions were prepared in Milli-Q water. The ^67^CuCl_2_ solution was purchased from Idaho Accelerator Center, Idaho State University. The Na^125^I solution was purchased from Perkin-Elmer. The fast protein liquid chromatography (FPLC) was performed on a GE ÄKTAFPLC system equipped with a Superdex 200 Increase 10/300 GL column and a Raytest in-line radio-detector. The mobile phase was 0.05 M phosphate buffer (pH 7.2) with 0.15 M NaCl and 2 mM ethylenediaminetetraacetic acid (EDTA). The flow rate was 1 mL/min. Radio-TLC analysis was performed on a Rita Star Radioisotope TLC Analyzer (Straubenhardt, Germany) to monitor the radiolabeling reaction using instant thin-layer chromatography (iTLC) paper and 1 × PBS as the developing solution.

### Cell cultures and animal models

All animal studies were performed in accordance with relevant guidelines and regulations through a protcol approved by the Institutional Animal Care and Use Committee (IACUC) at UT Southwestern, which adheres to the ARRIVE guidelines. Two cell lines (HCC1954 − HER2+ and MDA-MB-231 − HER2-) were used in this study. MDA-MB-231 cells was cultured in RPMI 1640 medium with 10% fetal bovine serum (FBS), 100 IU/mL penicillin, 100 μg/mL streptomycin, and 1% L-glutamine in a humidified incubator with 5% CO_2_ at 37 °C. All cell culture reagents were purchased from Invitrogen. The HCC1954 cell line were kindly provided by Dr. E. Sally Ward at UT Southwestern. The HCC1954 cell line was cultured in T-media (Invitrogen Corporation, CA) supplemented with 5% FBS and 1 × penicillin/streptomycin (PS). Female SCID mice (6–8 weeks of age) were purchased from the Wakeland Colony at UT Southwestern. To establish the tumor model, HCC1954 cell suspension was injected subcutaneously (0.5 × 10^6^ cells per injection with 50% BD matrigel, injection volume 100 µL) at the shoulder position. After injection, animals were monitored twice a week by general observations. The tumor volume was determined by the formula of Volume = (Width^2^ × Length)/2 by caliper measurements.

### Antibody conjugation

Conjugation of p-SCN-Bn-NOTA to pertuzumab was performed following previously reported methods^22^. Briefly, pertuzumab was firstly dialyzed to 0.05 M phosphate buffer (pH 7.2) with 0.15 M NaCl and then added a phosphate buffer (pH 8.5) containing 20-fold molar excess of p-SCN-Bn-NOTA. The resultant solution was stirred at room temperature for 3 h to form NOTA-Pertuzumab conjugate, which was subsequently purified by FPLC. The pooled fractions of NOTA-Pertuzumab conjugate were concentrated using Amicon Ultra 4 mL Centrifugal Filters (10 KDa cut-off) and in the same time the storage medium changed to 0.2 M ammonium acetate buffer (pH 6.5). The obtained immunoconjugate was stored at 4 °C for following uses. The protein concentration after modification was determined by the Coomassie (Bradford) Protein Assay.

### Analysis of the radiochemical purity of ^67^Cu

An aliquot (1 mL) of ^67^Cu was analyzed using a high purity germanium detector (HPGe) (Canberra, USA) for radiochemical impurities. The HPGe was calibrated for energy and efficiency using a 1 mL mixed gamma source (Eckert and Ziegler, USA). Identification of gamma rays was determined using the LBL gamma search database^48^.

### Radiolabeling

The radiolabeling of NOTA-Pertuzumab with ^67^Cu was typically carried out by adding 89 MBq (2.4 mCi) of ^67^CuCl_2_ in 0.1 M HCl to 3.2 µL of NOTA-Pertuzumab (25 µg/µL) pre-diluted with 0.05 mL 1 M NH_4_OAc buffer. The pH of the reaction medium was maintained at 5.0 ‒ 5.5 and the reaction was allowed to proceed for 30 min at 30 °C. The reaction mixture was sampled and then quenched with 5 µL of 5 mM diethylene triamine pentetic acid (DTPA) for 5 min at room temperature to determine the radiochemical yield via radio-iTLC, followed by radiochemical purity measurement by FPLC. The product, [^67^Cu]Cu-NOTA-Pertuzumab, obtained with > 99% radiochemical yield & purity was used for in vitro and in vivo studies. To determine the highest achievable radiochemical yield and specific activity, the ^67^Cu activity (27 MBq) and total reaction mixture volume were maintained the same while varying the amount of NOTA-Pertuzumab (0.5, 1, 2, 5, 25, 125 µg).

### In vitro stability

The in vitro stability test was performed in the rat serum. Briefly, [^67^Cu]Cu-NOTA-Pertuzumab (1.5 MBq, 5 μL) was added into 100 μL of rat serum (n = 3) and incubated at 37 °C. A 10 μL of sample was taken out and mixed with 2 μL of 5 mM DTPA from day 2 to day 7, respectively. The resulting solution was then analyzed by radio-TLC and radio-FPLC.

### Determination of average chelator number conjugated per antibody

The average number of NOTA chelators per pertuzumab molecule was determined by the isotope dilution method^49^. Briefly, a series of standardized ^nat^CuCl_2_ dilutions were prepared to react with the NOTA-Pertuzumab conjugate. For each ^nat^CuCl_2_ concentration, 10 μg (69 pmol) of the NOTA-Pertuzumab conjugate was added to 56 μL of 0.1 M NH_4_OAc, pH 5.0, followed by adding 40 μL of the standardized ^nat^CuCl_2_ solution and 1 μL of ^64^CuCl_2_ (4.4 MBq). The reaction mixture was incubated at 37 °C for 1 h, after which 10 μL of 5 mM DTPA was added to remove non-specifically bound ^64^Cu(II)/Cu(II). After 5 min, 1 μL of the reaction mixture was spotted for radio-TLC analysis. The plate was analyzed by a radio-TLC scanner. The number of chelators per antibody molecule was calculated based on a graph of the yield *vs.* the corresponding ^nat^CuCl_2_ concentration processed by GraphPad Prism 7.0 software^49^.

### Immunoreactivity

The immunoreactivity of [^67^Cu]Cu-NOTA-Pertuzumab was determined using a variation of the Lindmo assay in HER2+ HCC1954 cells^50^. Briefly, [^67^Cu]Cu-NOTA-Pertuzumab was diluted to 50 pg/μL in 1% BSA which corresponded to ~ 20,000 cpm/mL. A series of HCC1954 cell dilutions ranging from 1.25 × 10^5^ cells/mL to 2.5 × 10^6^ cells/mL were prepared and added to microfuge tubes, each containing 2.5 ng (2.8 kBq) of [^67^Cu]Cu-NOTA-Pertuzumab. After incubating the cells with gentle shaking at room temperature for 1 h, the microfuge tubes were centrifuged at 600×*g* for 2 min. After the supernatant removal, the cell pellets were washed with 500 μL of PBS. This centrifugation and washing procedure were repeated twice. The cell pellets were then counted using a PerkinElmer gamma counter. The total/bound activity versus 1/(normalized cell concentration) was graphed with GraphPad Prism 7.0, and the immunoreactive fraction was calculated by dividing 100 by the y-intercept obtained from the linear fit of the data.

### Western blotting and in vitro cell binding studies

HCC1954 (HER2+) and MDA-MB-231 (HER2-) were selected for cell studies. Firstly, western blot assay of HER2 was performed on both cell lines. Briefly, cell lysates (30 µg protein) were separated on a 4–12% Bis–Tris (NuPAGE) gel and transferred to a nitrocellulose membrane using an iBlot (Thermo Fisher Scientific) transfer system. Membranes were blocked with 3% milk in Tris-Buffered Saline Tween-20 (TBST) for 30 min and incubated overnight at 4 °C with the appropriate primary anti-HER2 antibody (Abcam, ab134182). After the membrane was washed for 15 min, the membrane was incubated with the secondary HRP donkey anti-rabbit antibody (ab6802) and developed with Supersignal West Pico ECL substrate (Thermo Fisher Scientific).

Cell uptake experiments were performed after the verification of positive HER2 expression in HCC1954 cells and negative HER2 expression in MDA-MB-231 cells. Cells were seeded in 24-well plates with 2.5 × 10^5^ cells per well and incubated overnight in a humidified incubator at 37 °C with 5% CO_2_. Cells were then incubated with 250 μL fresh complete media containing 25 ng/mL of [^67^Cu]Cu-NOTA-Pertuzumab. The plates were incubated at 25 °C for 1.5 h with gentle rocking. After incubation, the cells were washed with PBS in triplicate and then trypsinized. Trypsinized cells were placed in culture tubes and the radioactivity associated with the cells was counted. Bound radioactivity was calculated as the ratio of bound to the total radioactivity added per well.

### Radioimmunotherapy, toxicity assay

Radioimmunotherapy studies were performed on female SCID mice bearing HCC1954 tumor subcutaneously. When tumors grew to approximately 200 mm^3^ by average, the mice were randomized into 5 different groups and intravenously received 0.1 mg of trastuzumab 30 min prior to the tail vein injection of either the Pertuzumab only (Group ***1***) or [^67^Cu]Cu-NOTA-Pertuzumab (*Group 2*, ***3***, ***4***, and* 5*) (Table 2). Tumor growth was monitored with a digital caliper 2–3 times per week until the end of the study. Tumor volume (mm^3^) was calculated using the formula of ½ × (length × width^2^). The average difference in volume of the tumors was graphed *vs* the number of days after treatment in order to monitor the tumor response to therapy. The percent change in tumor volume over time was determined by the tumor size normalized to the beginning of treatment.

**Table 2:** Dosing information for control Group ***1*** and treatment Groups ***2***–***5***.

Group	Radioactivity Dose	Trastuzumab (µg)	Pertuzumab amount (µg)	[^67^Cu]Cu-NOTA-Pertuzumab specific activity (GBq/mg)	Number of mice
*1*	0	100	13.3	–	4
*2*	3.7 MBq (0.1 mCi)	100	3.3	1.1	6
*3*	7.4 MBq (0.2 mCi)	100	6.7	1.1	6
*4*	14.8 MBq (0.4 mCi)	100	13.3	1.1	7
*5*	7.4 MBq (0.2 mCi)	100	133.3	0.056	5

### SPECT/CT imaging

SPECT/CT imaging was performed at day 2 and day 5 post injection of [^67^Cu]Cu-NOTA-Pertuzumab on a NanoSPECT/CT Plus System (Bioscan, Washington, DC, USA). The field of view (FOV) of the SPECT/CT was adjusted to have the mouse in the center. CT imaging was performed using 360 projections per rotation with 45 kVp, 1000 ms exposure, and the binning factor of 1:4. The SPECT data were collected with 4 detector arrays collimated with multi-pinhole apertures giving a post-reconstruction resolution of 0.73 mm. SPECT images were acquired for 80 s in a standard anterior projection in list mode, at a rate of 1 frame per second. A calibration file was created using the known amount of ^67^Cu and then used to validate the following ^67^Cu imaging protocol. The SPECT imaging reconstruction was carried out using HiSPECT NG (Scivis wissenschaftliche Bildverarbeitung GmbH, Germany) with 35% smoothing, 100% resolution, and 3 × 3 iterations (Standard mode). The quantification of the ^67^Cu activity accumulated in tumor was performed using the Invicro’s VivoQuant 2.0 software package (Mediso, Boston, USA). After co-registration of the CT and SPECT images, a cylindrical region of interest (ROI) was drawn, encompassing the tumor and liver in all tomographic planes containing the organs. The total activity in the tumor was quantified as percentage injected dose per gram (%ID/g), which is defined as:

$$\% ID{\text{/g}} = \;\frac{{Activity\,\,\;\left( {\text{mCi/mL}} \right)}}{{Total\;Injected\;Dose\,\;\left( {{\text{mCi}}} \right)}} \times Density\; \times 100\quad \left( {assuming\;the\;density\;of\;tissue = 1\,{\text{g/mL}}} \right)$$

Although the NanoSPECT/CT system has a ^67^Cu acquisition protocol, the data analysis software lacks the ^67^Cu reconstruction file. Thus, a new ^67^Cu protocol was created from the In-111 protocol by modifying its energy detection range to 93 keV and 185 keV. A quantification calibration was performed using a 30 mL syringe filled with 29.6 MBq activity of ^67^Cu. The new quantification calibration file was added to the NanoSPECT/CT Plus isotope library for ^67^Cu reconstruction.

### Statistical analysis

Statistical analyses were performed using GraphPad Prism 7.0. A *p* value less than 0.05 (unpaired *t* test) was considered statistically significant. All results are presented as mean ± standard deviation.

## Supplementary Information

Supplementary figures.
