# Supplementary material for: Copper-67 radioimmunotheranostics for simultaneous immunotherapy and immuno-SPECT
Source: Sci Rep. 2021 Feb 11;11:3622. doi: 10.1038/s41598-021-82812-1 (PMC7878802; doi:10.1038/s41598-021-82812-1)
Supplement: Supplementary file 1 — Supplementary figures. [file 41598_2021_82812_MOESM1_ESM.docx]

**SUPPLEMENTARY INFORMATION**

**Copper-67 Radioimmunotheranostics for Simultaneous Immunotherapy and Immuno-SPECT**

Guiyang Hao^1^, Tara Mastren^1^, William Silvers^1^, Gedaa Hassan^1^, Orhan K. Öz^1^, and Xiankai Sun^1,2^

^1^Department of Radiology, ^2^Advanced Imaging Research Center, University of Texas Southwestern Medical Center, Dallas, Texas 75390, USA

Correspondence: Xiankai.Sun@UTSouthwestern.edu

**Contents**

**Supplementary Figures**

**Figure S1:** Full length western blots of HCC1954 and MDA-MB-231 cells ……………………………………………….. 2

**Figure S2:** Curves of tumor volume changes in each individual animal …………………………………………………… 3

**Figure S3:** Curves of body weight changes in each individual animal ………………………………………………………. 4

**Figure S4:** Gamma spectrum of ^67^Cu ……………………………………………………………………………………………………….. 4

**Figure S1**. Full length western blots of HER2 positive HCC1954 and HER2 negative MDA-MB-231 cells at different exposure levels. GAPDH was used as the loading control.

**Figure S2**. Curves of tumor volume (mm^3^) changes in each individual animal – **A**: Group 1, **B**: Group 2, **C**: Group 3, **D**: Group 4, and **E**: Group 5.

**Figure S3**. Curves of body weight changes in each individual animal of Group 1 – 5.


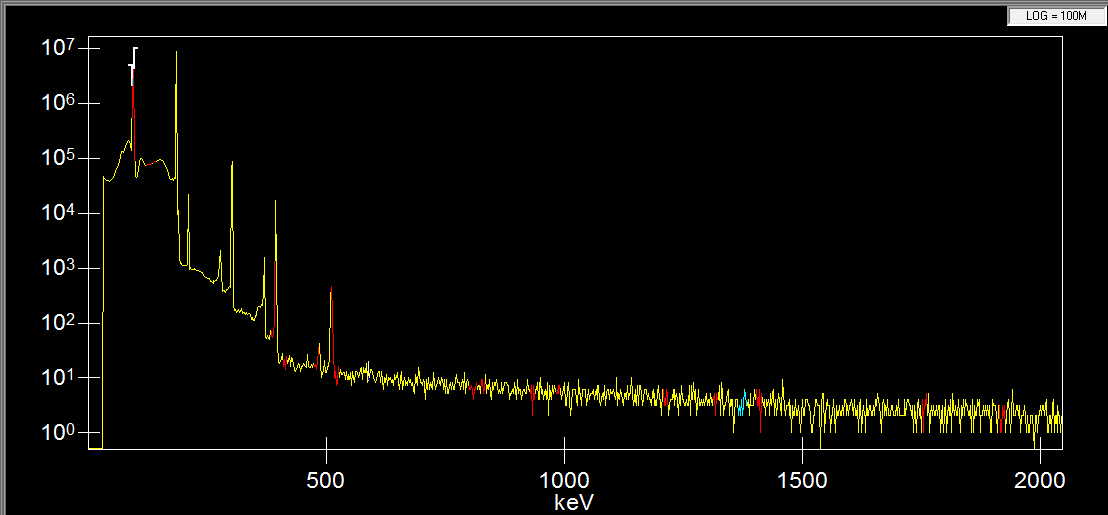


**Figure S4**. Gamma spectrum of ^67^Cu from a Canberra multichannel analyzer.
